# Examining pathways between family or peer factors and smoking cessation in a nationally representative US sample of adults with mental health conditions who smoke: a structural equation analysis

**DOI:** 10.1186/s12889-022-13979-z

**Published:** 2022-08-17

**Authors:** Catherine S. Nagawa, Bo Wang, Maryann Davis, Lori Pbert, Sarah L. Cutrona, Stephenie C. Lemon, Rajani S. Sadasivam

**Affiliations:** 1 Department of Population and Quantitative Health Sciences, University of Massachusetts Medical School, 368 Plantation Street, Worcester, MA 01605 USA; 2 Department of Psychiatry, University of Massachusetts Medical School, Worcester, MA USA; 3 Health Services Research & Development, Center of Innovation Edith Nurse Rogers Memorial Hospital Veterans Health Administration, Bedford, USA

**Keywords:** Smoking cessation, Mental health problems, Family and peer support

## Abstract

**Background:**

Supportive family or peer behaviors positively impact smoking cessation in people with mental health problems who smoke. However, the limited understanding of the pathways through which family or peer factors impact quitting limits the development of effective support interventions. This study examined pathways through which family or peer views on tobacco use, family or peer smoking status, and rules against smoking in the home influenced quitting in adults with mental health problems who smoke.

**Methods:**

We used data from the Population Assessment of Tobacco and Health Study, a national longitudinal survey. Baseline data were collected in 2015, and follow-up data in 2016. We included adults’ current smokers who had experienced two or more mental health symptoms in the past year (unweighted *n* = 4201). Structural equation modeling was used to test the relationships between family and peer factors, mediating factors, and smoking cessation.

**Results:**

We found that having family or peers with negative views on tobacco use had a positive indirect effect on smoking cessation, mediated through the individual’s intention to quit (regression coefficient: 0.19) and the use of evidence-based approaches during their past year quit attempt (regression coefficient: 0.32). Having rules against smoking in the home (regression coefficient: 0.33) and having non-smoking family members or peers (regression coefficient: 0.11) had a positive indirect effect on smoking cessation, mediated through smoking behaviors (regression coefficient: 0.36). All paths were statistically significant (*p* <  0.01). The model explained 20% of the variability in smoking outcomes.

**Conclusion:**

Family or peer-based cessation interventions that systematically increase intentions to quit and monitor smoking behavior may be able to assess the efficacy of family and peer support on quitting in people with mental health problems who smoke.

**Supplementary Information:**

The online version contains supplementary material available at 10.1186/s12889-022-13979-z.

## Introduction

Smoking remains the leading preventable cause of death globally, disproportionately affecting people with mental health problems [1]. Between 2008 and 2016, quit rates among people with mental health problems who smoke were consistently lower than quit rates in the general population each year, including most recently in 2016 (24% vs. 52%) [2]. There are several reasons for the high prevalence of smoking observed in individuals with mental health problems. For instance, psychological symptoms such as anxiety, low mood, or stress can trigger smoking. When smoking is used to reduce these symptoms, it may provide short-term relief, reinforcing the smoking behavior [3]. Until recently, it has not been the norm for mental health service providers to actively treat tobacco use [4]. Past exploratory qualitative studies conducted among people with mental health problems who smoke indicate that positive influences from family or peers facilitate successful quitting [5–7]. Supportive family or peer behaviors provide a strong incentive to quit [8], which may increase quitting intentions, enabling smoking cessation.

Family or peer-based interventions can be a practical approach to improving quit rates in people with mental health problems [5–7]. But much of the work has been conducted in the general population of people who smoke [9–14], and lacks evidence that the interventions achieved the aim of increasing the support provided to study participants [15, 16]. In people with mental health problems, there is scarce research that has explored how family and peer smoking status, rules on smoking in the home, and family or peer attitudes impact smoking cessation. Understanding the specific nature of these relationships can inform the development of support interventions that effectively address the cessation needs of people with mental health problems who smoke.

In the current study, we examined how family and peer factors influence smoking cessation using a nationally representative sample of people with mental health problems who smoke.

## Methods

### Study setting and data source

We used data from the Population Assessment Tobacco Health (PATH) study, a nationally representative, ongoing longitudinal study. PATH collects information on tobacco-use patterns, social influences, attitudes toward tobacco products, initiation, and cessation. Baseline data used in the current study were collected in 2015, and follow-up data in 2016 (one-year follow-up). Data were collected via computer-assisted personal interviewing and audio computer-assisted self-interviewing.

### Study sample

We included adults (≥ 18 years) who were currently smoking (had smoked at least 100 cigarettes in their lifetime and had smoked cigarettes in the past 30 days) at baseline and reported two or more mental health symptoms over the past year.

### Mental health symptoms

Mental health symptoms were measured using the Global Appraisal of Individual Needs Short Screener (GAIN-SS) [17]. The items for the GAIN-SS are derived from the full GAIN instrument, a validated and standardized biopsychosocial assessment for individuals entering treatment for behavioral health disorders [18, 19]. GAIN-SS is recommended for use in epidemiological samples [20], prior studies conducted among people living with mental health problems have used the GAIN-SS [21–23].

On the GAIN-SS scale, one score was assigned to each mental health symptom experienced over the past year. Scores range from zero to four on the internalizing disorder sub-scale and zero to seven on the externalizing disorder sub-scale. Participants can report up to eleven mental health symptoms on both subscales. Using clinically relevant cut points and as informed by past studies [21–23], we included participants who had experienced at least two mental health symptoms over the past year, regardless of the subscale. Validation studies indicate that those who report two or more symptoms are likely to have a mental health diagnosis [17].

**Fig. 1:**
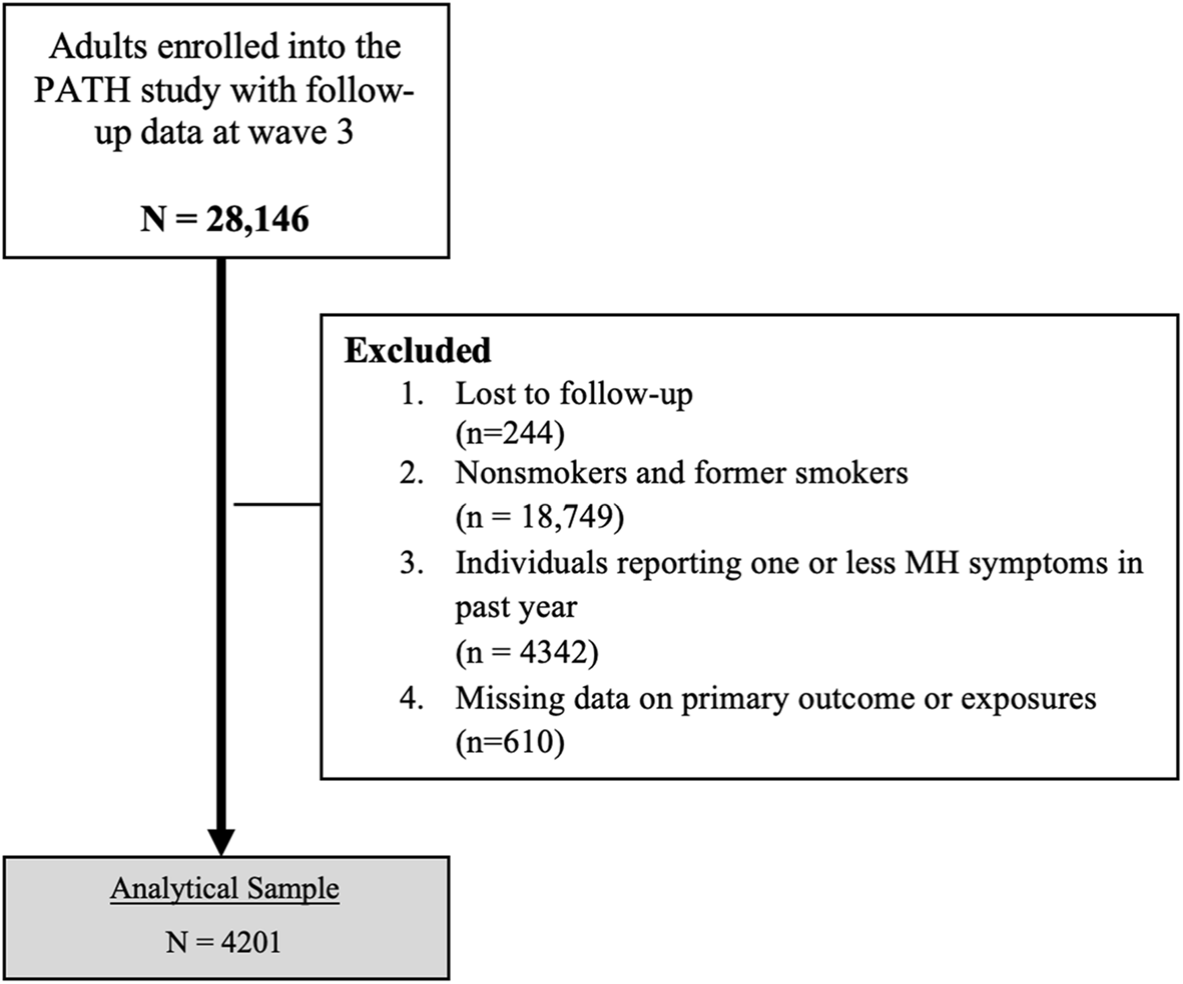
Exclusion criteria of the study population of people with mental health *problems* who smoke, Population Assessment of Tobacco and Health (PATH) Study

Among the 32,320 adults enrolled at baseline, 28,146 had complete data in 2015 (wave 3). We excluded 18,749 adults who were either nonsmokers or former smokers at baseline. We further excluded adult smokers who had not reported any mental health symptoms or reported only one symptom in the past year (*n* = 4342). Individuals who had missing data on primary exposures and outcomes (*n* = 610) were also excluded. Our analytical sample consisted of 4201 current adult smokers who had experienced two or more mental health symptoms over the past year (Fig. 1). Publicly available deidentified data were used in this study. Therefore, this research received an exemption from the institutional review board at the University of Massachusetts Chan Medical School.

### Measures

We collected data on sociodemographic factors (age, sex, race and ethnicity, marital status, whether participant lived alone, and education level), family and peer-related factors, intentions to quit smoking, the individual’s smoking behavior, self-perceived mental well-being, use of evidence-based cessation approaches, and smoking cessation. Data on demographic factors and exposure variables were measured at baseline (2015), and data on outcome variables were measured at a one-year follow-up (2016). Individual-level factors were measured at either baseline or follow-up. Timing details on individual-level factors are provided below (refer to the individual-level factors section).

#### Exposure variables of interest

1. *Family or peer views on tobacco use:* Participants were asked to report family or peer views on tobacco use, using the question, “Thinking about the people who are important to you, how would you describe their views on using tobacco in general?” Response options: very positive, positive, neutral, negative, very negative.
2. *Family or peer smoking status.* The smoking status of those who were important to them was captured using the question, “Thinking about the people who are important to you, do any of them use cigarettes?” (Responses were yes, no).
3. *Rules on smoking in the home:* This was captured using the question; “For tobacco products that are burned, such as cigarettes, cigars, pipes, or hookah, which statement best describes the rules about smoking a tobacco product inside your home?” Participants responded by indicating whether 1) smoking was not allowed anywhere or anytime, 2) smoking is allowed in some places or sometimes, or 3) Smoking is allowed anywhere, at any time.

#### Individual-level factors

**Fig. 2:**
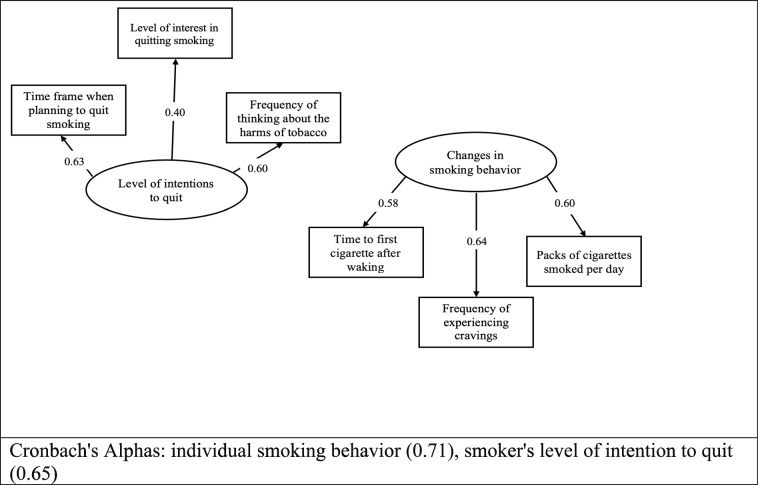
Confirmatory factor analysis of the smoker’s level of intention to quit and the individual smoking behavior of the study participants

4. *Intentions to quit smoking*: Intentions to quit were assessed using three measures in which participants reported, 1) levels of interest in quitting (measured at baseline), 2) the time frame within which they planned to quit smoking (measured at baseline), and 3) how frequently they thought about the harms associated with using tobacco (measured at follow-up). All three measures are strongly associated with the smokers’ intentions to quit [24], and have practical applications when distinguishing between individuals with low and those with high intentions to quit smoking in the stages of change behavioral model [24, 25]. Level of interest in quitting was measured using the statement “Overall, on a scale from 1 to 10 where one is not at all interested and ten is extremely interested, how interested are you in quitting smoking cigarettes? Please choose a number from 1 to 10” In the second measure of intentions to quit, participants were asked to indicate, on a scale of 1 to 5, the time frame within which they planned to quit smoking (1 = In the next 7 days, 2 = In the next 30 days, 3 = In the next 6 months, 4 = In the next year, 5 = More than 1 year from now). The third measure captured how frequently smokers thought about the harm associated with using tobacco, using the question, “In the past 30 days, how often did you think about the harm your tobacco use might be doing to you?” Participants responded on a scale of 1 to 5 (1 = Never, 2 = Rarely, 3 = Sometimes, 4 = Often, 5 = Very often). Cronbach’s alpha for the intentions to quit smoking scale was 0.65 (Fig. 2).
5. *Smoking behaviors:* We described the smoking behavior of participants using three variables: number of cigarettes smoked per day (measured at baseline), time to the first cigarette after waking (measured at baseline), and cigarette cravings (measured at baseline), which are all behavioral markers for tobacco dependence [26–29] Number of cigarettes was assessed as packs smoked per day. Time to the first cigarette after waking was assessed using the question, “How soon after you wake up do you smoke your first cigarette? 1 = Within 5 minutes, 2 = 6 to 30 minutes, 3 = 31 to 60 minutes, 4 = After 60 minutes. Higher values indicated a lowered dependence on cigarette smoking. Participants also rated their level of agreement to the following statement to capture the frequency of cigarette cravings, “I find myself reaching for tobacco products without thinking about it” on a scale of 1 = not true of me at all to 5 = extremely true of me. Cronbach’s alpha for the smoking behavior scale was 0.71 (Fig. 2).

6. *Self-perceived mental well-being:* Participants’ self-perception of their mental health was assessed using the question. “In general, how would you rate your mental health, which includes stress, depression, and problems with emotions?” Responses included 1 = Excellent, 2 = Very good, 3 = Good, 4 = Fair, 5 = Poor. Self-perceived mental health was reverse coded in the analysis such that higher values corresponded to better self-perceived mental well-being.
7. *Use of evidence-based cessation approaches during past year’s quit attempt(s)*: This variable was assessed using a two-stage process. First, participants were asked, “In the past 12 months have you tried to quit smoking/using tobacco product(s)?” (measured at follow-up). Participants responded with either a yes or no. Those who had made a past-year quit attempt were then asked if they had used evidence-based cessation approaches during the quit attempt. Use of evidence-based cessation strategies during past-year quit attempts was assessed using four questions: 1) “In the past 12 months, have you used counseling, telephone helpline, books, pamphlets, videos, quit tobacco clinic, class, support group, or web-based program to help when you last tried to quit smoking?”?“ (measured at follow-up), 2) “In the past 12 months, have you used a nicotine patch, gum, inhaler, nasal spray, lozenge, or pill when you last tried to quit smoking?“ (measured at follow-up), and 3) “Thinking back to the time you tried to quit in the past 12 months, did you use Chantix, varenicline, Wellbutrin, Zyban, or bupropion?” (measured at follow-up). Participants responded with either yes or no on each of the three questions. We then classified them into three groups; 1 = those who had made a past-year quit attempt(s) using any of the evidence-based cessation approaches, 2 = those who had made a past-year quit attempt(s) but had not used any of the evidence-based cessation approaches, and 3 = those who had not made a past-year quit attempt.

#### Outcome variable of interest

8. *Smoking Cessation.* We assessed current smoking status at the one-year follow-up, using the question, “Do you currently smoke cigarettes (1 = every day, 2 = somedays, and 3 = not at all)?)

### Formulation of the hypothesized model

Having the perception that immediate family or peers disapprove of one’s smoking is associated with making a quit attempt [8, 30]. We, therefore, hypothesized that having family or peers with negative views on tobacco use had a direct relationship with smoking cessation and an indirect relationship mediated through intentions to quit and smoking behaviors. Intentions reflect the extent to which individuals are motivated to perform a behavior and are conceptualized as the most proximal antecedent of behavior [31]. Thus, having higher intentions to quit was hypothesized to be associated with using evidence-based cessation approaches during quit attempt (s), which in turn was hypothesized to be associated with smoking cessation.

**Fig. 3:**
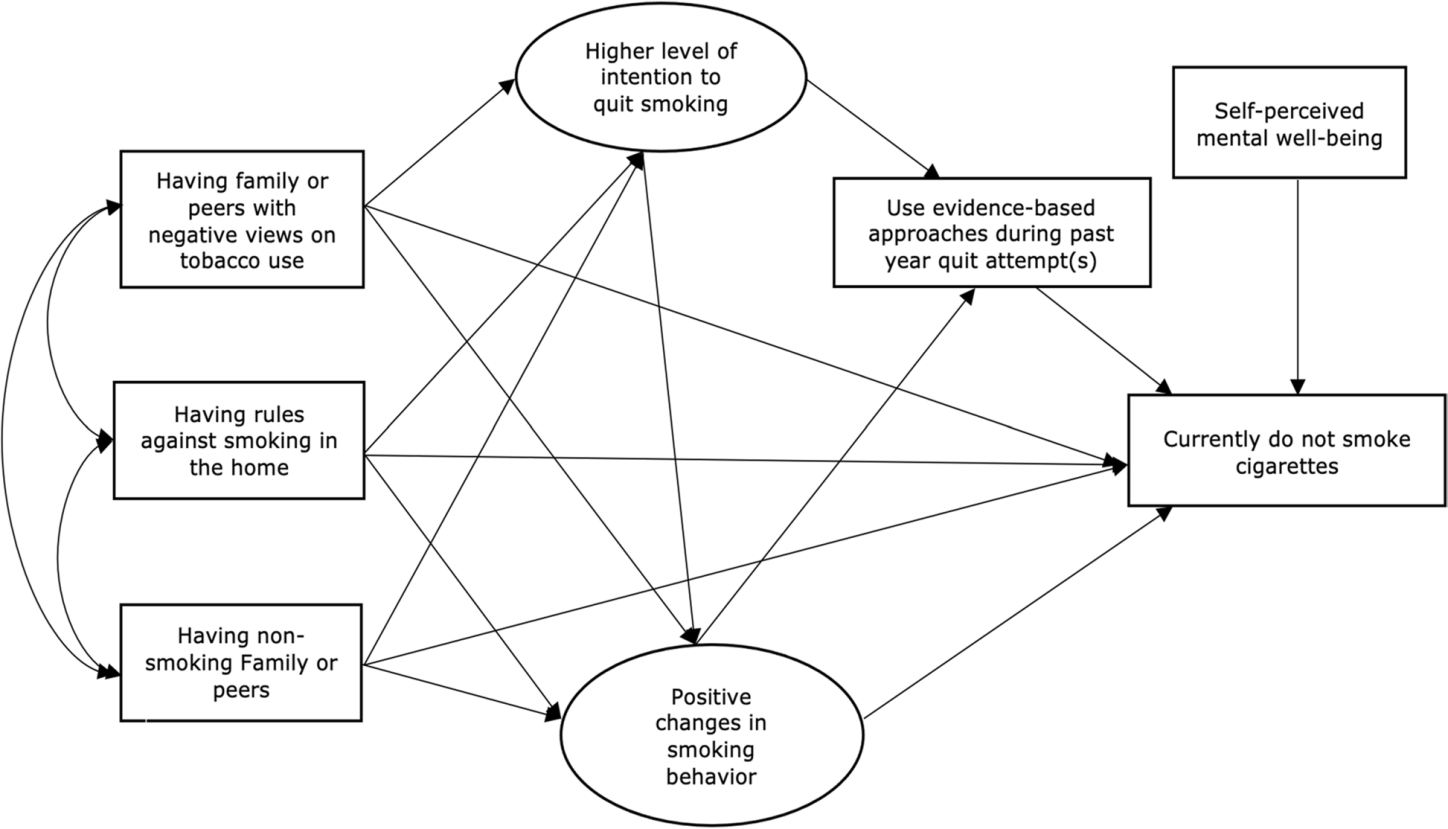
Hypothesized relationships between family or peer factors and smoking cessation in smokers with mental health problems

Successful quitters tend to have non-smoking families or peers [32] and rules against smoking in the home [33]. We hypothesized that having rules against smoking in the home and non-smoking family members or peers would directly or indirectly affect smoking cessation in adults with mental health problems who smoke. We also assessed self-perceived mental well-being as a covariate in the relationship between family and peer factors (views, rules, and smoking status of family or peers) and smoking cessation (Fig. 3).

### Statistical analysis

Spearman correlation analysis was conducted to examine the strength of correlations between variables. To account for the complex sampling procedures of the PATH study, we obtained weighted correlations using the ‘corr_svy’ command in STATA, which displays correlation coefficients that account for the probability sampling weights.

The analysis was a two-step process. First, we used confirmatory factor analysis to form latent variables from indicator variables. Descriptive analysis, confirmatory factor analysis, and correlation analysis were performed in STATA (V.15). We then constructed a structural model to test the hypothesized relationships between latent constructs and manifest variables. We used Wald tests criteria to remove non-significant paths that did not increase the model chi-square. The model was evaluated using the goodness-of-fit index (GFI), chi-square to degrees-of-freedom (df) ratio, root mean square error of approximation (RMSEA), and Tucker-Lewis index (TLI). Good model fit is determined by an RMSEA less than 0.08, chi-square to degrees-of-freedom (df) ratio less than 5, and values of GFI and TLI greater than 0.90. Structural model procedures were conducted using the Mplus statistical software package (V 7.0) We used the Sobel test to test the significance of the mediating effect of one’s intentions to quit and smoking behaviors in the association between family and peer influences on smoking cessation. The Sobel test is a commonly used method for testing the significance of the mediation effect [34].

## Results

### Participant characteristics

Forty-eight percent of respondents were male (48%), and one in four (25%) were aged between 25 and 35. A majority (71%) self-identified as non-Hispanic White, and 11% as non-Hispanic Black. About half (51%) had a high school education level or less. The average number of cigarettes smoked per day was 13.4 (SD: 28.8). The mean number of mental health symptoms was five (mean: 5.4; SD 3.1) (Table 1**).**

**Table 1:** Weighted percentage distributions of participant characteristics of smokers with mental health problems, using data from the Population Assessment of Tobacco Health Study (2015–2016)

Participant characteristics	Weighted %
Age	
18 to 24	17.3
25 to 34	25.4
35 to 44	20.0
45 to 54	19.5
55 and older	17.9
Men	47.9
Race/ethnicity	
Non-Hispanic White	70.9
Non-Hispanic Black	11.3
Hispanic	9.8
Other	7.9
Education attainment	
High school or less than high school	51.3
Marital status	
Married	31.9
Widowed/Separated/Divorced	30.8
Never married	37.2
Do you currently live alone?	
No	82.1
Mental health symptoms, mean (SD)	5.4 (3.1)
Cigarettes smoked per day, mean (SD)	13. 4 (28.8)

### Correlation between family or peer views on tobacco use, rules against smoking in the home, family or peer smoking status, and smoking cessation in adults with mental health problems who smoke

All three family and peer factors were positively correlated with each other. Having family or peers with negative views on tobacco use positively correlated with having rules against smoking in the home (correlation coefficient: 0.17, *p*-value < 0.01) and having non-smoking family members or peers (correlation coefficient: 0.23, *p*-value < 0.01). Having rules against smoking positively correlated with having non-smoking family or peers (correlation coefficient: 0.14, *p*-value < 0.01).

Having family or peers with negative views on tobacco use positively correlated with higher intentions to quit (correlation coefficient: 0.19, *p*-values < 0.01). Higher intentions to quit positively correlated with the use of evidence-based cessation approaches during a past year-quit attempt (s) (correlation coefficient: 0.33, *p*-value < 0.01). Using evidence-based cessation approaches during a past-year quit attempt was positively correlated with no current use of cigarettes (correlation coefficient: 0.41, *p*-value < 0.01).

Rules against smoking in the home positively correlated with positive smoking behaviors (correlation coefficient 0.33; *p*-value < 0.01) and no current use of cigarettes (correlation coefficient: 0.16; *p*-value). Having a non-smoking family or peers also positively correlated with positive smoking behaviors (correlation coefficient: 0.12; *p*-value < 0.01). Positive smoking behaviors correlated with no current use of cigarettes (correlation coefficient: 0.36, *p*-value < 0.01) (Supplementary Table 1).

### Structural equation model fit

The initial hypothesized model tested is shown in Fig. 2, included 17 paths. Using the Wald test criteria, we removed non-significant paths (*p*-value greater than 0.05) that did not increase the model chi-square. Self-perceived mental well-being did not have a significant path with any of the other variables (see supplementary Table 1); therefore, we excluded this variable from the final model. The resulting final model had a good fit; RMSEA was 0.04, the TLI was 0.95, and the CFI was 0.97. All paths shown in the final model were statistically significant (standardized coefficients are shown in Table 2). The overall model explained 20% of the variability in smoking cessation outcomes.

**Table 2:** The coefficients and the corresponding 95% Confidence Interval and *P*-values between variables in the model

Variables included in final model	Estimate	Standard Errors	95% Confidence Interval	***P***-value
Currently do not smoke cigarettes ➞ Use of evidenced-based approaches during a past year’s quit attempt	0.33	0.014	0.301 – 0.349	< 0.001
Currently do not smoke cigarettes ➞ Smoking behaviors	0.36	0.014	0.334 – 0.381	< 0.001
Use of evidenced-based approaches during a past year’s quit attempt ➞ Intentions to stop smoking	0.32	0.016	0.294 – 0.346	< 0.001
Intentions to stop smoking ➞ Family or peer views on tobacco use	0.19	0.016	0.163 – 0.217	< 0.001
Family or peer views on tobacco use ➞ Family or peers who smoke	0.23	0.017	0.202 – 0.258	< 0.001
Family or peer views on tobacco use ➞ Rules against smoking in the home	0.16	0.017	0.132 – 0.188	< 0.001
Family or peer views on tobacco use ➞ Smoking behavior	0.07	0.017	0.043 – 0.097	< 0.01
Rules on smoking in the home ➞ Smoking behavior	0.33	0.017	0.306 – 0.354	< 0.001
Family or peers who smoke ➞ Smoking behavior	0.11	0.018	0.083 – 0.137	< 0.001
Rules on smoking in the home ➞ Family or peers who smoke	0.13	0.017	0.103 – 157	< 0.001

### Associations between family or peer factors, smoking behaviors, and smoking cessation

**Fig. 4:**
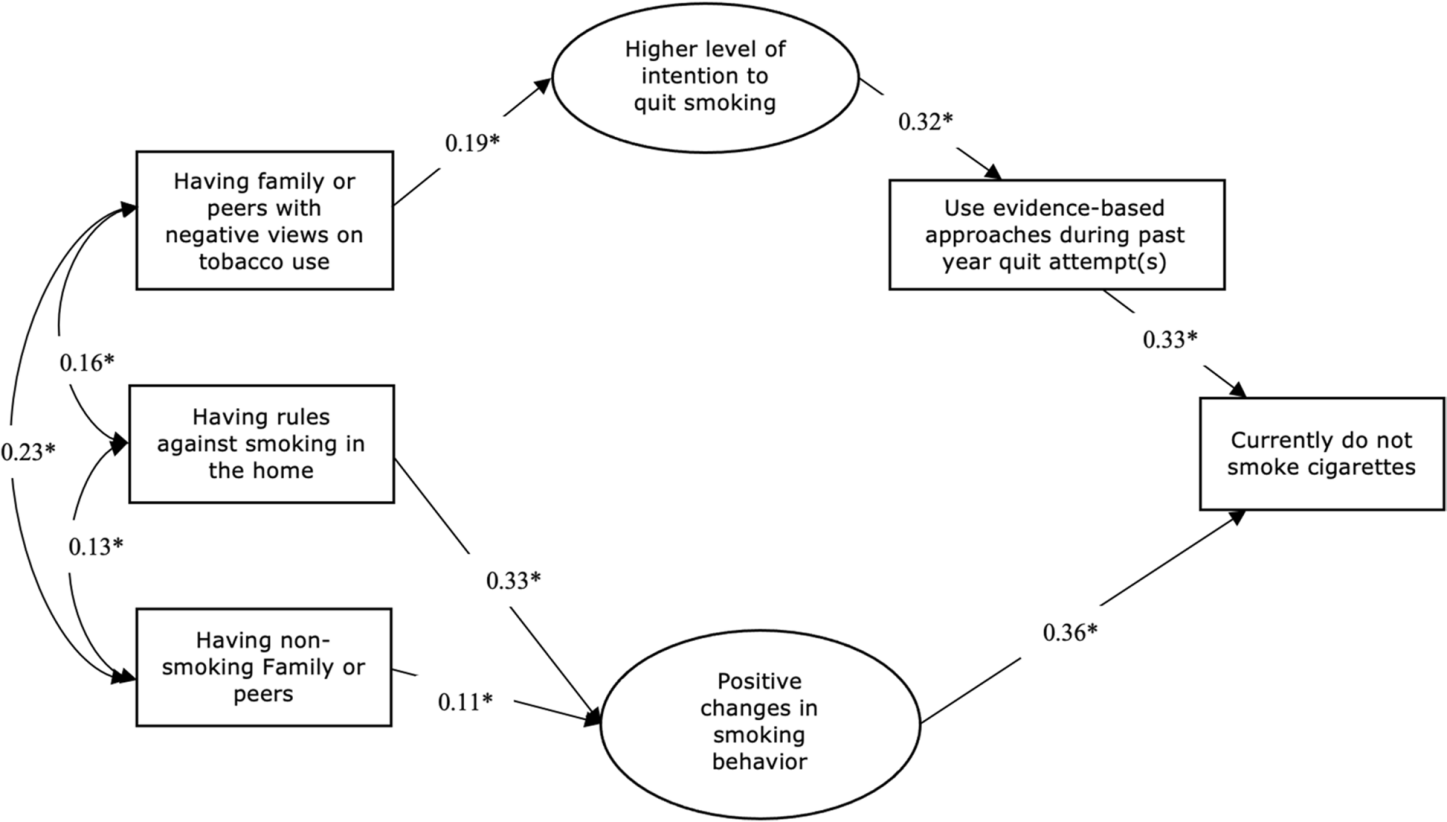
Final model depicting relationships between family or peer factors and smoking cessation in smokers with mental health problems using data from the Population Assessment of Tobacco and Health (PATH) Study (2015–2016)

Family and peer negative views on tobacco use had an indirect effect on smoking cessation, mediated through the smoker’s intention to quit (regression coefficient: 0.19), which was associated with using evidence-based smoking cessation approaches during a past year quit attempt (regression coefficient: 0.32). Using evidence-based approaches during a past year attempt was associated with smoking cessation (regression coefficient: 0.33). Having rules against smoking in the home (regression coefficient: 0.33) and having non-smoking family members or peers (regression coefficient: 0.11) had positive indirect effects on smoking cessation, mediated through the individual’s smoking behavior (regression coefficient: 0.36) (Fig. 4).

The Sobel test of mediation effect indicated that family or peers’ negative views on tobacco use had an indirect relationship with smoking cessation, mediated through intentions to quit (z = 10.2, *p*-value = < 0.001) and having rules against smoking in the home and non-smoking family or peers had an indirect relationship with smoking cessation, mediated through smoking behavior (non-smoking family or peers; z = 5.5, *p*-value = < 0.01; having rules against smoking; z = 15.8, *p*-value = < 0.01)).

## Discussion

We aimed to identify the pathways through which family or peer factors influenced smoking cessation using a nationally representative US sample of adults with mental health problems who smoke. We identified two paths: 1) family or peers’ negative views on tobacco use had an indirect relationship with smoking cessation, mediated through intentions to quit and use of evidence-based approaches during a past year quit attempt, and 2) having rules against smoking in the home, and non-smoking family or peers had an indirect relationship with smoking cessation, mediated through smoking behavior.

Our findings showed that family or peers who held negative views on tobacco use positively influenced smoking cessation by increasing one’s intentions to quit and promoting the use of evidence-based cessation approaches during quit attempts. This finding is consistent with past research that illustrates the positive benefits of a supportive social environment for motivating smokers to quit [35–37]. However, misconceptions about smoking and mental health symptom management are common among family members and peers [38, 39] and tend to undermine quitting success. A proposed strategy is to use educational programs to change family and peer views on tobacco use. Cessation interventions that partner with family or peers could also regularly monitor for changes in intentions to quit and use of evidence-based approaches to correctly evaluate the efficacy of family and peer influences on quitting in this population.

Having rules against smoking in the home and non-smoking family members or peers were both associated with the desired smoking behaviors (including a reduction in the number of cigarettes smoked and experiencing cravings less frequently), which promoted quitting one year later. Past research shows that smoking-related cravings are often produced by pairing an external stimulus, such as holding a cigarette, with access to nicotine [40, 41]. Therefore, supportive family or peers’ behaviors can positively impact quitting by reducing the number of smoking cues in the individual’s physical environment. Given that a majority (82%) of participants in our study lived with someone, people who smoke may be more likely to follow smoking rules in the home if there is someone to be accountable to. Implementing smoke-free rules and encouraging quitting in significant others who smoke could improve quit rates in this population. Furthermore, regular monitoring of smoking behaviors (such as reduction in the number of cigarettes smoked or frequency of experiencing cravings) in behavioral interventions could provide evidence of family and peer support on quitting success in people with mental health problems who smoke.

All three family or peer behaviors and attitudes investigated in this study were associated with each other but worked through distinct behavioral routes to influence smoking status. That is, having rules against smoking in the home and non-smoking family and peers improved smoking behaviors, and negative views on tobacco use boosted the smoker’s motivation to quit. This finding is consistent with research on social norms [42], which indicates that descriptive social norms (what other people do) and injunctive norms (what other people think you should do) can independently influence behavior [43, 44]. Cessation interventions that aim to alter multiple family or peer factors could significantly improve quit rates in people with mental health conditions who smoke.

### Limitations

This study had several limitations that need to be kept in mind when interpreting the results. The GAIN-SS measures the severity of mental health symptomatology and does not provide a diagnosis. However, the high sensitivity and specificity between GAIN-SS items and clinical diagnosis tools [17] support the use of symptoms as good indicators of clinically significant mental health conditions [20]. Second, the model focused on the effects of family and peer factors on smoking cessation. We did not account for other factors that may impact cessation outcomes in this population, such as access to mental health services [45]. Third, our study is limited by its lack of information from family or peers. Dyadic views from family, peers, and people who smoke are equally informative when developing support interventions. We faced a data limitation were mediator variables (intentions to quit smoking, smoking behaviors, and use of evidence-based cessation approaches during past year’s quit attempts) were collected at either baseline or follow-up, and not at the mid-point. Nonetheless, the use of structural equation modeling allowed for a better understanding of the links between the different factors and the mechanisms of their association.

## Conclusion

When examined simultaneously, family and peer factors indirectly affected smoking cessation. Changing existing family or peer norms on tobacco use is necessary to facilitate successful quitting. Our study indicates that different aspects of family and peer support correlate and may work through different pathways to influence smoking behaviors. Family or peer-based cessation interventions that systematically improve mediators identified in this study may be able to assess the efficacy of family and peer support on quitting in people with mental health problems who smoke.

## Supplementary Information

**Additional file 1: Supplementary Table 1.** Correlations between family or peer factors, self-perceived mental well-being, smoking behavior, and smoking cessation in smokers with mental health conditions using data from the Population Assessment of Tobacco Health Study (2015–2016).

## Data Availability

The datasets generated and analyzed during the current study are available in the National Addiction & HIV Data Archive Program repository, https://www.icpsr.umich.edu/web/NAHDAP/studies/36231.
