# Supplementary material for: Examining pathways between family or peer factors and smoking cessation in a nationally representative US sample of adults with mental health conditions who smoke: a structural equation analysis
Source: BMC Public Health. 2022 Aug 17;22:1566. doi: 10.1186/s12889-022-13979-z (PMC9382825; doi:10.1186/s12889-022-13979-z)
Supplement: Supplementary file 1 — Additional file 1: Supplementary Table 1. Correlations between family or peer factors, self-perceived mental well-being, smoking behavior, and smoking cessation in smokers with mental health conditions using data from the Population Assessment of Tobacco Health Study (2015–2016). [file 12889_2022_13979_MOESM1_ESM.docx]

**Supplementary Table 1:** Correlations between family or peer factors, self-perceived mental well-being, smoking behavior, and smoking cessation in smokers with mental health conditions using data from the Population Assessment of Tobacco Health Study (2015 – 2016)

| Latent and Manifest Variables | 1 | 2 | 3 | 4 | 5 | 6 | 7 | 8 |
| --- | --- | --- | --- | --- | --- | --- | --- | --- |
| 1. no current use of   cigarettes | 1.00 |  |  |  |  |  |  |  |
| 1. use of evidence-based   cessation approaches | 0.41* | 1.00 |  |  |  |  |  |  |
| 1. high level of intention   to quit | 0.07* | 0.32* | 1.00 |  |  |  |  |  |
| 1. smoking   behavior | 0.36* | 0.08* | 0.03 | 1.00 |  |  |  |  |
| 1. having family/peer negative views on tobacco use | 0.04 | 0.04 | 0.19* | 0.07* | 1.00 |  |  |  |
| 1. rules against smoking in   the home | 0.16* | 0.05* | 0.10* | 0.33* | 0.17* | 1.00 |  |  |
| 1. self-perceived mental   well-being | -0.01 | 0.04 | 0.05* | -0.07* | -0.04 | 0.01 | 1.00 |  |
| 1. non-smoking family   or peers | 0.07* | 0.01 | 0.06* | 0.12* | 0.23* | 0.14* | -0.05* | 1.00 |
| *p value < 0.01 | | | | | | | | |
